# Long-Term Alteration of Intestinal Microbiota in Patients with Ulcerative Colitis by Antibiotic Combination Therapy

**DOI:** 10.1371/journal.pone.0086702

**Published:** 2014-01-29

**Authors:** Shigeo Koido, Toshifumi Ohkusa, Takayuki Kajiura, Junko Shinozaki, Manabu Suzuki, Keisuke Saito, Kazuki Takakura, Shintaro Tsukinaga, Shunichi Odahara, Toyokazu Yukawa, Jimi Mitobe, Mikio Kajihara, Kan Uchiyama, Hiroshi Arakawa, Hisao Tajiri

**Affiliations:** 1 Division of Gastroenterology and Hepatology, Department of Internal Medicine, The Jikei University School of Medicine, Tokyo, Japan; 2 Frontier Research Laboratories, Institute for Innovation, Ajinomoto Co., Inc., Kawasaki, Japan; 3 Pharmaceutical Laboratories, Ajinomoto Co., Inc., Kawasaki, Japan; CWRU/UH Digestive Health Institute, United States of America

## Abstract

Previous work has demonstrated that intestinal bacteria, such as *Fusobacterium varium* (*F. varium*), contribute to the clinical activity in ulcerative colitis (UC); thus, an antibiotic combination therapy (amoxicillin, tetracycline, and metronidazole (ATM)) against *F. varium* can induce and maintain UC remission. Therefore, we investigated whether ATM therapy induces a long-term alteration of intestinal microbiota in patients with UC. Patients with UC were enrolled in a multicenter, randomized, double-blind, placebo-controlled study. Biopsy samples at the beginning of the trial and again at 3 months after treatment completion were randomly obtained from 20 patients. The terminal restriction fragment length polymorphism (T-RFLP) in mucosa-associated bacterial components was examined to assess the alteration of the intestinal microbiota. Profile changes of T-RFLP in mucosa-associated bacterial components were found in 10 of 12 patients in the treatment group and in none of 8 in the placebo group. Dice similarity coefficients using the unweighted pair group method with arithmetic averages (Dice-UPGMA) confirmed that the similarity of mucosal microbiota from the descending colon was significantly decreased after the ATM therapy, and this change was maintained for at least 3 months. Moreover, at 3 months after treatment completion, the *F. varium*/β-actin ratio, examined by real-time PCR using nested PCR products from biopsy samples, was reduced less than 40% in 8 of 12 treated patients, which was higher, but not significantly, than in 4 of 8 patients in the placebo group. Together, these results suggest that ATM therapy induces long-term alterations in the intestinal microbiota of patients with UC, which may be associated, at least in part, with clinical effects of the therapy.

## Introduction

The etiology of ulcerative colitis (UC) is still unknown; however, increasing evidence suggests that the intestinal microbiota play important roles in the pathogenesis of inflammatory bowel disease (IBD), including: a) intestinal microbiota adhere to the crypt epithelium via Toll-like receptors (TLR)2 and TLR4 and then invade the epithelium, resulting in inflammatory cytokine production; b) intestinal microbiota are necessary for the development of spontaneous colitis and immune system activation in IL-2- and IL-10-deficient mice; and c) an overly aggressive cell-mediated immune response to endogenous intestinal bacterial constituents in genetically susceptible patients results in intestinal inflammation [1]–[4]. Although the immune cells in the intestinal lamina propria balance the requirements for immune tolerance of microbiota with defense against microbial pathogens [5], IBD is described as a state of excessive inflammation in response to bacterial microbiota antigens leading to impaired function of the intestinal barrier [6]–[8]. Moreover, the intestinal microbiota profile varies considerably between active UC patients and healthy controls [2]. Therefore, appropriate antibiotic therapy against microbial pathogens could alter the intestinal microbiota in patients with UC, resulting in the improvement and remission of active UC.

Previously, we have reported that *Fusobacterium varium* (*F. varium*) was present in the colonic mucosa of a high proportion (84%) of UC patients [9] and that butyric acid, a product of *F. varium* culture supernatants, caused UC-like lesions in mice [10]. Therefore, we recently conducted a double-blind placebo-controlled multicenter trial to determine whether an antibiotic combination regimen (amoxicillin, tetracycline, and metronidazole (ATM) for 2 weeks) against the microbial pathogens of UC, including *F. varium*, induces and/or maintains remission of active UC [11]. The ATM therapy produced an improvement of endoscopic and Mayo scores, remission, and steroid withdrawal in active UC more effectively than a placebo. Moreover, among the patients positive for *F. varium* antibodies, the clinical response rate was significantly higher in the ATM group than in the placebo group vs. the placebo group [11]. That study indicated that *F. varium*, at least in part, is associated with the UC condition. In this study to assess the alteration of intestinal microbiota by the ATM therapy, biopsy samples that were randomly obtained from the clinical trial were analyzed.

We show that the profile of the terminal restriction fragment length polymorphism (T-RFLP) in the mucosa-associated bacterial components is significantly changed at 3 months after the ATM therapy. Dice similarity coefficients using the unweighted pair group method with arithmetic averages (Dice-UPGMA) cluster analysis confirms the alteration of the intestinal microbiota after the ATM treatment. While the *F. varium*/β-actin ratio in the intestinal microbiota, as analyzed by real-time PCR from nested PCR products, is reduced after the ATM treatment, the reduction is not significant compared with the placebo group.

## Materials and Methods

### Study subjects

A multicenter, randomized, double-blind, placebo-controlled study was conducted between January 2004 and July 2006 at 11 hospitals in Japan [11]. This trial was registered at http://www.umin.ac.jp/ctr/index-j.htm:UMIN000000078 (Newly developed antibiotic combination therapy for ulcerative colitis: a double-blind placebo-controlled multicenter trial). All eligible patients had an established diagnosis of UC. Study subjects were selected from among patients with chronic relapsing UC (at least one relapse per year) with more than 1 year of follow-up and regular visits to outpatient clinics. The eligibility criteria for study entry were mild-to-severe disease with a colonoscopy score of at least 1 (erythema, decreased vascular pattern, and mild friability) based on a scale of 0 (normal or inactive) to 3 (spontaneous bleeding and ulceration), and/or watery diarrhea at least 5 times/day, with visible blood in stools. Patients with toxic megacolon or a penicillin allergy, and those who were pregnant, had serious liver or renal disease or any psychological illness, were excluded. Patients who had taken antibiotics within 4 weeks before study entry or had *Clostridium difficile* or other stool pathogens (*Salmonellae*, pathogenic *Escherichia coli*, *Campylobacter jejuni*, *Shigellae*, etc.) at entry were also excluded.

### Study design

We recruited 210 patients and randomly assigned them to either the ATM or the control placebo group in the clinical trial [11]. The ATM treatment group (oral amoxicillin 1500 mg/day, tetracycline 1500 mg/day, and metronidazole 750 mg/day) and the placebo group each had 105 subjects. Each group was treated for 2 weeks and were then followed up. Twenty subjects in these clinical trials were randomly selected for this study. The study used central randomization with a treatment allocation produced by a computer-generated randomization list. Among the 20 patients, 12 patients were in the antibiotic treatment group and 8 were in the placebo group. Therapy was started after initial endoscopy at entry. The follow-up endoscopy was carried out 3 months after treatment to determine whether the antibiotic combination therapy induces and/or maintains the remission of active UC. Biopsy samples were taken from the cecum, ascending colon, and descending colon at the beginning of the trial and again at 3 months after treatment completion. The biopsy samples were immediately placed in formalin for histopathological assessment or in liquid nitrogen for storage at −80°C until the subsequent isolation of DNA. All histopathology, clinical evaluations, and endoscopies were carried out by physicians who were blinded to the patients' therapy. Clinical assessments were evaluated by the symptom score of Lichtiger et al. [12]. Endoscopic-and histological scores were graded according to the Matts score [13]. Histological scores were the sum of the scores of the cecum, ascending colon, transverse colon, and descending colon. The study protocol was reviewed and approved by the ethics committee of the Juntendo institutional review board, Juntendo University School of Medicine, as well as the clinical study committee of the Juntendo University Hospital (No. 2003-016). All subjects gave written informed consent. All authors had access to the study data and have reviewed and approved the final manuscript.

### Isolation of DNA

DNA from each biopsy sample (the cecum, ascending colon, and descending colon) was extracted using the UltraClean Soil DNA Isolation Kit (Mo Bio Laboratories, Solana Beach, CA, USA) with some modifications [14]. Briefly, each biopsy sample was washed by gently shaking in sterile water to remove loosely adherent fecal material and mucus. The samples were placed in 2 mL Bead Solution tubes containing 550 µL Bead Solution and mixed by vortexing on a FastPrep instrument (Bio 101, Vista, CA, USA) for 10 s at 4 m/s, followed by the addition of lysozyme (final concentration of 1 mg/mL) and N-acetylmuramidase (final concentration of 20 µg/mL) and incubation at 37°C for 1 h. After incubation, 60 µL of Solution S1 and 200 µL of Solution IRS (inhibitor removal solution) were added and the samples were vortexed on a FastPrep for 10 s at 4 m/s. The supernatant was collected by centrifugation at 12,000 g for 5 min, mixed with Solution S2, vortexed gently, and placed on ice for 5 min. The mixture was centrifuged at 13,000 rpm for 1 min and the supernatant was transferred to a clean microcentrifuge tube. After the addition of 900 µL of Solution S3, the sample was gently vortexed. The mixture was loaded onto a spin filter tube and centrifuged at 12,000 g for 1 min. The flow-through solution was discarded, 300 µL Solution S4 was added, and the spin filter was centrifuged at 13,000 rpm for 1 min. The flow-through solution was discarded and the spin filter was placed in a new tube. Approximately 50 µL of Solution S5 was added, and after the sample was centrifuged at 13,000 rpm for 1 min, the spin filter was discarded and the DNA was collected in the tube.

### Nested-PCR

The primers used for PCR amplification of 16S ribosomal DNA (rDNA) sequences were 27F (5′-AGAGTTTGATCCTGGCTCAG-3′) and 1492R (5′-GGTTACCTTGTTACGACTT-3′) [15], [16]. The secondary primers were 529F (5′-ACGTGCCAGCAGCCGCCG-3′) and 1492R. Primer 529F was labeled at the 5′-end with 6′-carboxyfluorescein (6-FAM), which was synthesized by Sigma-Aldrich Japan K.K. (Tokyo, Japan). Amplification reactions were performed in a total volume of 50 µL containing 5 µL dissolved DNA (100 ng), 1.25 U TaKaRa Ex Taq (Takara Shuzo, Shiga, Japan), 5 µL of 10X Ex Taq buffer, 4 µL dNTP mixture (2.5 mM each), and 10 pmol of each primer. The 16S rDNAs were amplified in a thermal cycler (Gene Amp PCR System 9700; Applied Biosystems, Foster City, CA, USA) using the following program: 95°C for 3 min, followed by 30 cycles consisting of 95°C for 30 s, 50°C for 30 s, and 72°C for 1.5 min, with a final extension period at 72°C for 10 min. The PCR reaction mixture and the PCR cycling conditions in the second round of nested-PCR were the same. Amplified DNA was verified by electrophoresis of aliquots of PCR mixtures (2 µL) on 1.5% agarose gels in 1× TAE buffer. The PCR products were purified using the polyethylene glycol (PEG) precipitation method [17], with some modifications. A 50 µL aliquot of the 16S rDNA solution was mixed with 30 µL of PEG solution (40% PEG 6000 and 10 mM MgCl_2_) and 12 µL of 3 M sodium acetate and then centrifuged at 15,000 rpm for 15 min. The supernatant was carefully removed by pipetting, and then the precipitated DNA was washed twice with 70% ethanol and was redissolved in 20 µL sterile distilled water. Negative controls for PCR consisted of sterile water used in place of the DNA template, and they were used in parallel for every round of PCR preparation.

### Terminal restriction fragment length polymorphism (T-RFLP) analysis from nested-PCR products

The purified nested-PCR products (2 µL) were digested with 20 U of *Hha*I (Takara Shuzo) in a total volume of 10 µL at 37°C for 3 h. The restriction digestion product (1 µL) was mixed with 12 µL of deionized formamide and 1 µL of DNA fragment length standard. The standard size marker was a 1∶1 mixture of the size standard GS 500 ROX (including 35, 50, 75, 100, 139, 150, 160, 200, 300, 350, 400, 450, 490 and 500 bp) and GS 1000 ROX (including 29, 33, 37, 64, 67, 75, 81, 108, 118, 244, 275, 299, 421, 539, 674, 677 and 926 bp) (Applied Biosystems). Each sample was denatured at 95°C for 2 min and then immediately placed on ice. The length of the TRF was determined on an ABI PRISM 3130 genetic analyzer (Applied Biosystems) in GeneMapper mode (15 kV, 8 mA and 60°C for 48 min for each sample). Fragment sizes were estimated using the Local Southern Method in Genescan 3.1 software (Applied Biosystems).

### Cluster analysis of T-RFLP profiles

Mucosal microbiota from the ATM treatment group and the placebo group were collected at the beginning of the trial and again at 3 months after treatment completion. Cluster analysis of mucosal microbiota from the descending colon was performed by Dice similarity coefficients using the unweighted pair group method with arithmetic averages (Dice-UPGMA) [18].

### Real-time PCR for quantification of *F. varium* DNA

Nested-PCR products were used to quantify *F. varium* DNA because the sample amounts were extremely small. The primer sets for *F. varium* were designed as previously reported [19], [20]. The primer sets for *F. varium* were designed by aligning their 16S rDNA gene sequences (obtained from GenBank database) with those from closely related bacteria using the ClustalW program [20]. First, 16S rDNA was amplified using the 27F and 1492R primers [15], [16], and then the DNA was amplified using the primer sets specific for 16S rDNA sequences in *F. varium*. The primer sets for 16S rDNA sequences in *F. varium* were (5′-CCGTTGCTTATATGGGGTTG-3′) and (5′-CCTCGCAGATTCACACAGAA-3′). The primer set for human β-actin was purchased from Maxim Biotech Inc. (South San Francisco, CA, USA). The primer set sequences for human β-actin were (5′-GCCAACCGCGAGAAGATGACC-3′) and (5′-CTTTAGCACGCACTGTAATTCCTC-3′). DNA isolated from biopsy samples (the cecum, ascending colon, and descending colon) was amplified with species-specific primers and the primer set for human β-actin. PCR was performed in a 50 µL volume containing 2.5 µL of the DNA sample, 25 µL of SYBR Green PCR Master Mix (Applied Biosystems) and 15 pmol of each primer. Amplification and detection were performed with a sequence detection system (ABI PRISM 7900HT; Applied Biosystems). The protocol for *F. varium* was 50°C for 2 min, 95°C for 10 min, and then 50 cycles at 95°C for 15 s and 60°C for 1 min. The protocol for human β-actin was 50°C for 2 min, 95°C for 10 min, and then 50 cycles at 95°C for 30 s and 60°C for 1 min. The number of bacterial cells or human β-actin gene copies in tissue samples was estimated with a standard curve for each species or for the human β-actin gene. The standard curves were drawn using the results from five concentrations of bacterial cells (10^7^, 10^6^, 10^5^, 10^4^ and 10^3^ cells/µL) or the human β-actin gene (10^6^, 10^5^, 10^4^, 10^3^ and 10^2^ copies/µL). Finally, the results of real-time PCR for each sample were expressed as the number of bacterial cells per 10^6^ copies of the human β-actin gene.

### Statistical analysis

Statistical analysis was performed using Student's *t*-test and Fisher's exact test for statistical comparison of clinical, histological, and endoscopic grades, and the number of bacterial cells at the trial start and 3 months after treatment completion. STAT VIEW software, version J 5.1 (SAS Institute, Inc., Cary, NC, USA) was used for all analyses. *P*<0.05 was considered significant.

### Ethical considerations

This study, UMIN000000078, was registered at http://www.umin.ac.jp/ctr/index-j.htm. The institutional review board or ethics committee approved the protocol.

## Results

### Characterization of enrolled patients with UC

**Figure 1:**
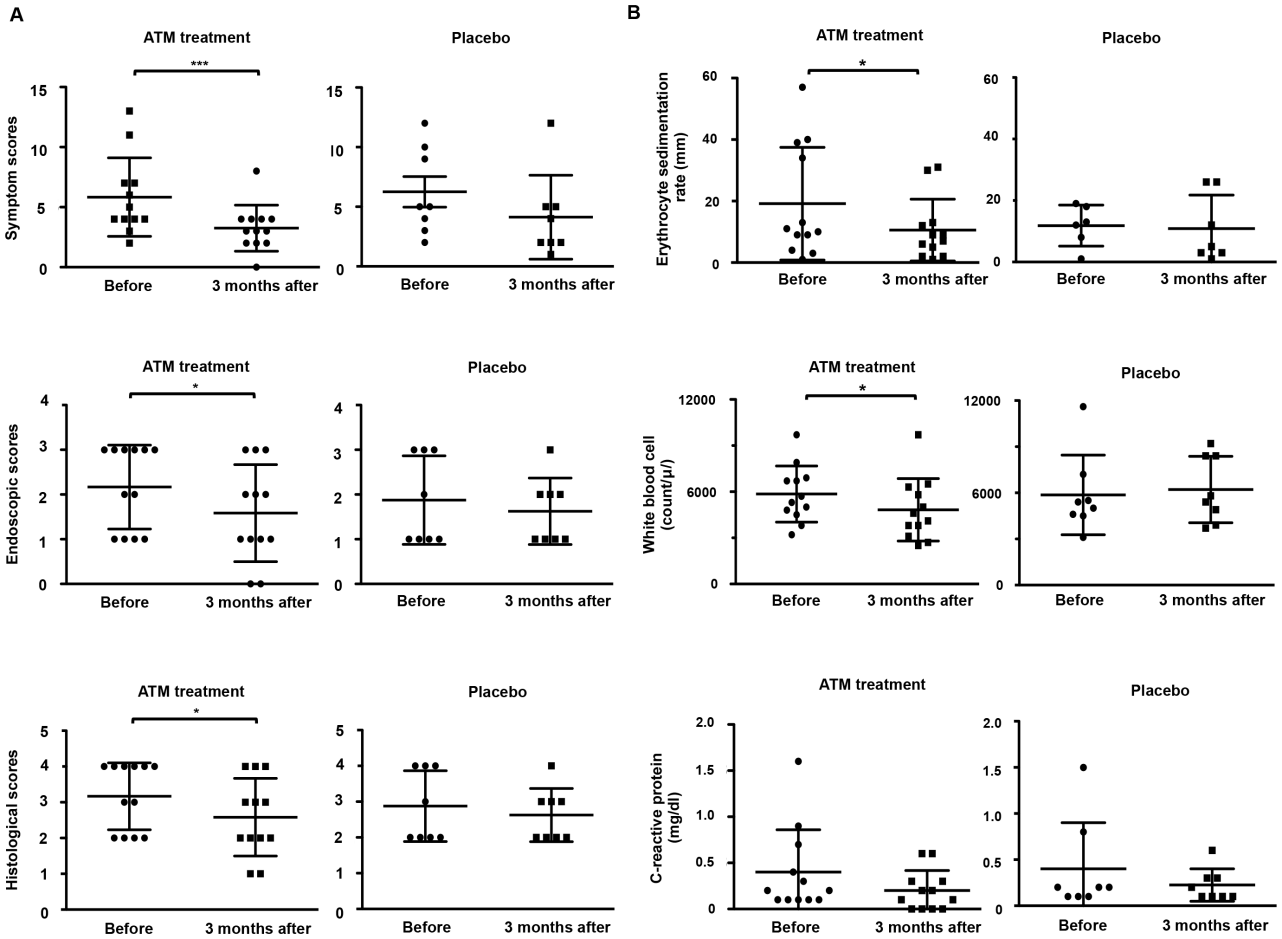
Characterization of the patients with UC. Patients with UC were treated with the ATM therapy (n = 12) or placebo (n = 8) for 2 weeks. (A) The symptom score (upper panel), endoscopic score (middle panel), and histological score (lower panel) at 3 months after the ATM treatment or placebo were compared with the trial start. (B) Inflammatory markers such as the erythrocyte sedimentation rate (upper panel), white blood cell count (middle panel), and C-reactive protein (CRP) (lower panel) at 3 months after the ATM treatment or placebo were compared with the trial start. The results are expressed as the mean ± SD. ***P<0.001; **P<0.01; *P<0.05.

Biopsy samples (the cecum, ascending colon, and descending colon) were obtained from 20 patients with UC in the randomized clinical study that had demonstrated the significant clinical effects of the ATM therapy (n = 105) compared with placebo (n = 105) (*P* = 0.0011) [11]. The 20 patients included 12 patients from the ATM treatment group and 8 patients from the placebo group. There were no differences in age, gender, mean duration of disease, current activity, extent of disease, or concomitant medications between the ATM treatment and placebo groups (Table 1). Before the treatment, there were also no differences in the symptom [12], endoscopic, and histological scores between the ATM treatment and placebo groups [13] (Table 2). The symptom, endoscopic and histological scores at 3 months after the ATM treatment were significantly improved compared with study entry (*P* = 0.0033, 0.0463 and 0.0463, respectively) (Fig. 1A). Moreover, at 3 months after the ATM treatment, inflammatory markers, such as the ESR and WBC, were significantly decreased compared with study entry (*P* = 0.0226 and 0.0495, respectively) (Fig. 1B). C-reactive protein (CRP) was also reduced at 3 months after the ATM treatment, but not significantly, compared with study entry (*P* = 0.0580). At 3 months after the ATM treatment, the symptom, endoscopic, and histological scores were also improved in 25% (3/12), 50% (6/6), 41.7% (5/12) of the patients, respectively, which was higher, but not significantly, than the placebo group (12.5% (1/8), 25% (2/8), 12.5% (1/8), respectively). However, among the 20 patients randomly enrolled in this study, there were no significant differences in the symptom, endoscopic, histological scores or the inflammatory markers at 3 months after treatment completion between the ATM group and the placebo group (Table 2). In addition, no serious drug-related toxicity was observed during the trial (data not shown).

**Table 1:** Clinical characterization of patients with ulcerative colitis in antibiotics and placebo groups.

Characteristics	Antibiotic group (n = 12)	Placebo group (n = 8)	*p*-value
Mean age, year (range)	39.75 (27–69)	44.0 (18–62)	*P*>0.2
Gender (male/female)	7/5	7/1	*P*>0.2
Mean duration of disease, year (range)	7.1 (1–20)	6.4 (1–21)	*P*>0.2
Current activity (mild/moderate/severe)	5/7/0	4/3/1	*P*>0.2
Extent of disease, number of patients (%)			*P*>0.2
Pan colitis	3 (25)	2 (25)	
Left-sided colitis	6 (50)	4 (50)	
Proctitis	3 (25)	2 (25)	
Concomitant medications during study			
Number of patients (%)			*P*>0.2
Sulfasalazine	8 (66.7)	5 (62.5)	
5-ASA	4 (33.3)	3 (37.5)	
Glucocorticoid	3 (25.0)	2 (25.0)	

5-ASA, mesalazine.

**Table 2:** Symptom score, endoscopic finding score, histological score, and inflammatory markers in antibiotics and placebo groups.

Variable	Antibiotic group (n = 12)	Placebo group (n = 8)	*P*-value
Mean symptom score (range)			
at entry	5.8 (4–13)	6.3 (2–13)	*P*>0.2
at 3 months	3.3 (0–8)	4.1 (1–12)	*P*>0.2
Mean endoscopic finding score (range)			
at entry	2.2 (1–3)	1.9 (1–3)	*P*>0.2
at 3 months	1.6 (0–3)	1.6 (1–3)	*P*>0.2
Mean histological score (range)			
at entry	3.2 (2–4)	2.9 (2–4)	*P*>0.2
at 3 months	2.6 (1–3)	2.6 (2–4)	*P*>0.2
ESR (range)			
at entry	19.2 (1–57)	11.8 (1–19)	*P*>0.2
at 3 months	10.6 (1–30)	10.86 (1–26)	*P*>0.2
CRP (range)			
at entry	0.4 (0.1–1.6)	0.4 (0.1–1.5)	*P*>0.2
at 3 months	0.1 (0–0.3)	0.2 (0.1–0.6)	*P* = 0.072
WBC (range)			
at entry	5850 (3200–9700)	5863 (3100–11600)	*P*>0.2
at 3 months	5517 (3100–9000)	6213 (3700–9200)	*P*>0.2

Symptom grades were scored according to Lichtiger et al.'s symptom score. Endoscopic and histological findings were scored according to Matts's grading score. ESR: erythrocyte sedimentation rate (mm). CRP: C-reactive protein (mg/dl). WBC: white blood cell (count/µl).

### T-RFLP analysis

**Figure 2:**
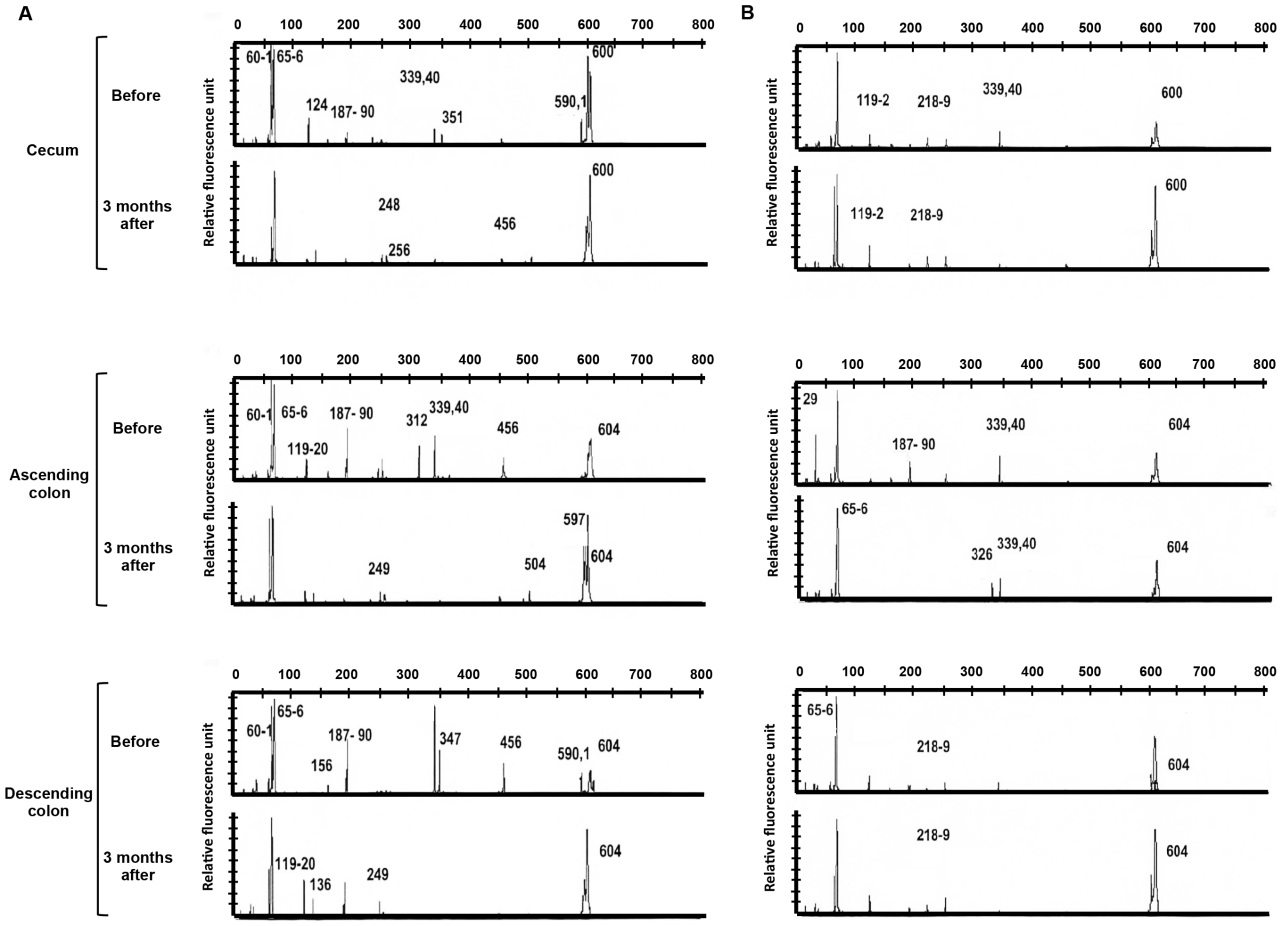
Representative T-RFLP patterns of 16S rDNAs from mucosal bacteria. (**A**) There were no characteristic peak patterns in the ATM treatment groups. At 3 months after treatment completion, the peak heights of the restriction enzyme *Hha*I were changed in the ATM therapy group (A) but not changed in the placebo group (B).

The 16S ribosomal RNA (rRNA) gene is highly conserved between different species of bacteria and archaea. Therefore, the 16SrRNA gene has been used as the standard for classification and identification of microbes. The genes coding for it are referred to as 16S rDNA. Thus, to avoid contamination by human mucosal DNA, the 16S rDNA was amplified by PCR in all 20 patients. T-RFLP analysis of the PCR amplified product was used to characterize the differences in mucosal flora from UC patients in the antibiotic treatment group (n = 12) and the placebo group (n = 8). The peaks of each T-RF length (bp) show the relative quantity of 16S rDNA fragments for each T-RF length. There were no characteristic peak patterns in UC between the antibiotic treatment group (Fig. 2A) and the placebo group (Fig. 2B). Interestingly, a sub-set of the fragments for the restriction enzyme *Hha*I changed after treatment in 10 of 12 patients (83.3%) in the antibiotic treatment group, but no changes were observed in the placebo group (*P* = 0.0007). These results indicate bacteria whose 16S rDNA fragments change length after restriction by *Hha*I were reduced by antibiotic treatment.

### Cluster analysis of T-RFLP profiles

**Figure 3:**
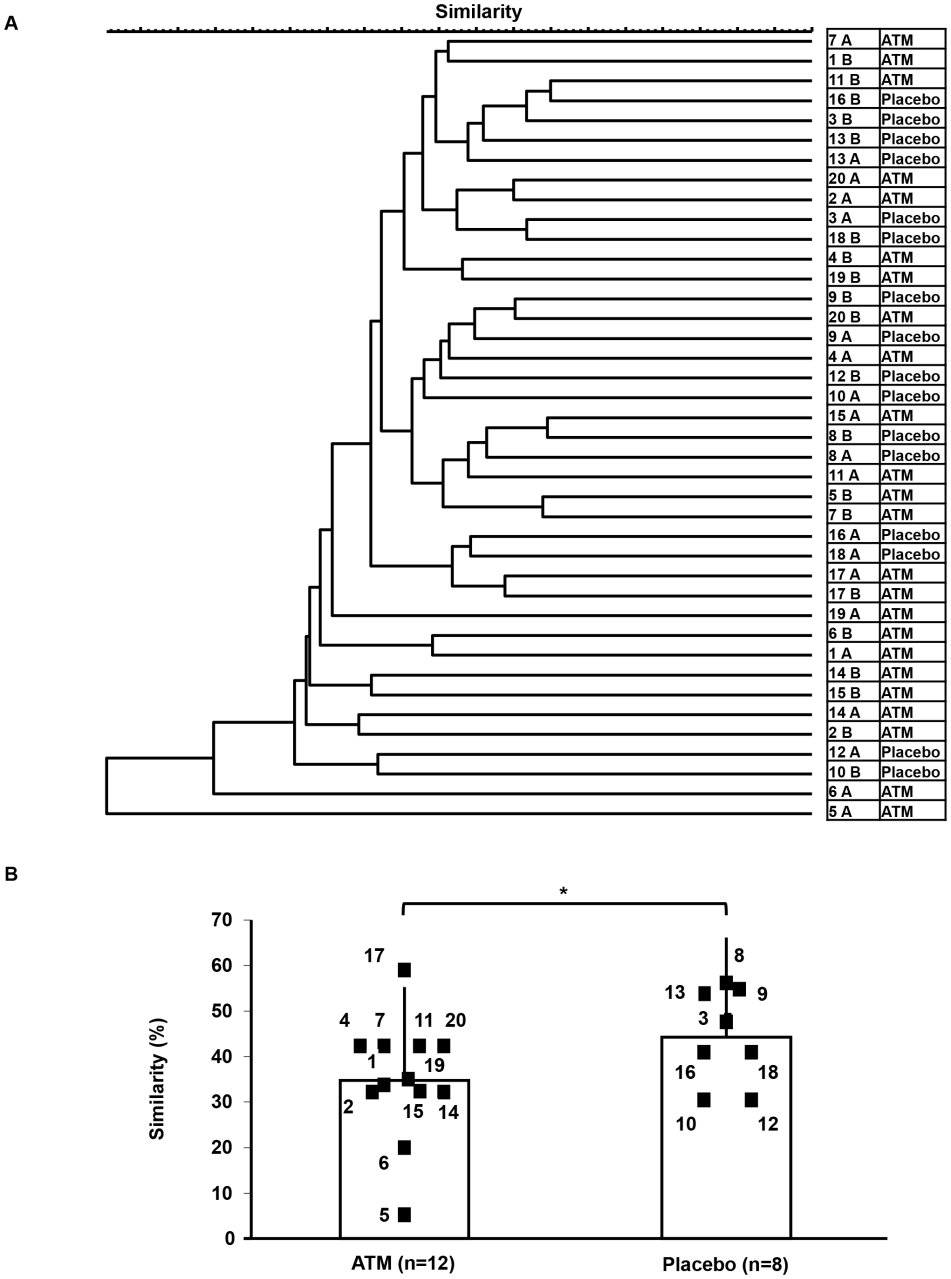
Dendrogram of T-RFLP profiles before and after the ATM therapy. (A) The similarity dendrogram was constructed using a binary coefficient dendrogram (Dice-UPGMA). The numbers indicate the subject code number in the ATM and placebo groups. A; after therapy, B; before therapy. (B) Similarity values from the dendrogram at trial start and again at 3 months after treatment completion (ATM or placebo) were analyzed. The results are expressed as the mean ± SD. *P<0.05.

Three months after treatment, the similarity of mucosal microbiota was significantly decreased in the ATM group (35.0%) compared to the placebo group (44.5%) by Dice-UPGMA cluster analysis (*P* = 0.0454) (Fig. 3A and B). This result indicates that ATM therapy can induce and maintain significant alterations of intestinal microbiota in patients with UC at least 3 months after the treatment.

### Quantitative analysis of *F. varium*

To analyze *F. varium* quantitatively in the mucosal flora after ATM treatment, real-time PCR from nested-PCR products was used, as clinically obtained biopsy sample amounts were usually extremely small. In the clinically obtained biopsy samples, there are the variations in biopsy size, and DNA extracts of the biopsies have a preponderance of human nucleic acid relative to bacterial nucleic acid. The human β-actin gene has frequently been used as an internal control gene to normalize the expression of the target mRNA levels between different samples measured by real-time PCR. Therefore, we standardized comparison of the numbers of bacteria associated with biopsies by determining the ratio of *F. varium* equivalents to the number of human β-actin equivalents. In this study, the numbers of *F. varium* in each sample (the cecum, ascending colon, and descending colon) per 10^6^ copies of the human β-actin gene were examined. Based on this method, the total number of the *F. varium*/β-actin ratio in each sample (the cecum, ascending colon, and descending colon) was 40% reduced after treatment in 8 of 12 patients (66.7%) in the ATM group, which was higher, but not significantly, than 4 out of 8 patients (50.0%) in the placebo group (*P*>0.05) (Table S1).

## Discussion

The data presented here show that the 2-week triple-antibiotic therapy produced a long-term alternation of the intestinal microbiota that persisted at least 3 months compared to a placebo in a multicenter, randomized, double-blind, placebo-controlled study.

To analyze the alteration of mucosa-associated bacteria after the ATM therapy, we first used T-RFLP analysis. In this study, significant profile changes of the T-RFLP in the mucosa-associated bacterial components were found in 10 of 12 patients in the ATM treatment group, compared with none of 8 patients in the placebo group; therefore, ATM therapy is strongly associated with the alteration of mucosa-associated bacteria in patients with UC. UPGMA analysis also confirmed the significant alteration of mucosal microbiota at least 3 months after the ATM therapy. Importantly, an alteration of the intestinal microbiota may cause a breakdown of the barrier function, such as an abnormal immune response to normal flora, and result in UC [21]–[26]. Therefore, long-term alterations in the intestinal microbiota produced by ATM therapy may be associated with improvements in UC. However, among the 20 patients with UC analyzed in this study, there were no differences in the symptom, endoscopic and histological scores or in the inflammatory markers at 3 months after treatment completion between ATM and placebo groups. The small sample size used in this study may be associated with this contradiction. Indeed, our 20 subjects were from a randomized clinical trial that demonstrated significant clinical effects of the ATM therapy (n = 105) compared with a placebo (n = 105) [11].

Our previous research demonstrated that *F. varium* was one of the causative agents in UC [9], [10]. Therefore, we also analyzed *F. varium* quantitatively in the mucosal flora of UC patients treated with ATM. In this study, samples were taken from the cecum, ascending colon, and descending colon at the beginning of the trial and again at 3 months after treatment completion. Because extremely small amounts were obtained in the clinical trial; we tried to quantify *F. varium* by real-time PCR from nested-PCR products in the mucosal flora. We demonstrated that the *F. varium*/β-actin ratio in the ATM treatment group tended to decrease, but not significantly, compared with the placebo group. In contrast, a decreased number of *F. varium* in UC patients treated by the ATM therapy was reported in a previous study [24]. However, the decreased number of *F. varium* in that study was analyzed by real-time PCR using samples taken from the appendix, caecum, ascending colon, transverse colon, descending colon, sigmoid colon, and rectum. Therefore, analysis using the nested PCR products from small amounts of biopsy specimens may not be suitable for quantifying *F. varium* in UC patients. Other pathogens besides *F. varium* may also be associated with the pathogenesis of UC. As these three antibiotics used for the ATM therapy are lethal to many bacterial species, unidentified pathogens could be more significantly decreased by the ATM therapy.

In conclusion, the mucosa-associated bacterial components analyzed by T-RFLP are useful to assess the alteration of the intestinal microbiota in UC after ATM therapy. The ATM antibiotic combination regimen significantly altered intestinal microbiota in UC patients compared to the placebo group. Our study supports the association of microbial agents with the pathogenesis of UC. A limitation of our study is the relatively small sample size. Further studies are needed to evaluate the clinical significance of long-term alteration of intestinal microbiota in UC patients treated with ATM.

## Supporting Information

Table S1 (DOCX)
