# Supplementary material for: Long-Term Alteration of Intestinal Microbiota in Patients with Ulcerative Colitis by Antibiotic Combination Therapy
Source: PLoS One. 2014 Jan 29;9(1):e86702. doi: 10.1371/journal.pone.0086702 (PMC3906066; doi:10.1371/journal.pone.0086702)
Supplement: Table S1 — (DOCX) [file pone.0086702.s001.docx]

**Table S1. Alternations of the total number of *F. varium*/β-actin after treatment**

| Patient No | Treatment | Biopsy sampling lesions | Study entry | 3 months after treatment | After/Before (%) * |  |
| --- | --- | --- | --- | --- | --- | --- |
|  |  |  |  |  |  |  |
| 1 | ATM | Cecum-Appendix | 0.216 | 0.020 |  |  |
|  |  | Ascending-Transverse | 0.172 | 0.784 |  |  |
|  |  | Descending-Rectum | 0.723 | 0.696 |  |  |
|  |  | Total ** | 1.111 | 1.500 | 135.014 |  |
| 2 | ATM | Cecum-Appendix | 0.136 | 2.375 |  |  |
|  |  | Ascending-Transverse | 0.957 | 1.630 |  |  |
|  |  | Descending-Rectum | 3.311 | 0.300 |  |  |
|  |  | Total | 4.404 | 4.305 | 97.752 |  |
| 3 | Placebo | Cecum-Appendix | 1.059 | 0.060 |  |  |
|  |  | Ascending-Transverse | 1.341 | 0.656 |  |  |
|  |  | Descending-Rectum | 1.318 | 0.963 |  |  |
|  |  | Total | 3.718 | 1.679 | 45.159 |  |
| 4 | ATM | Cecum-Appendix | 0.833 | 0.738 |  |  |
|  |  | Ascending-Transverse | 3.214 | 0.957 |  |  |
|  |  | Descending-Rectum | 2.044 | 0.581 |  |  |
|  |  | Total | 6.091 | 2.276 | 37.367 |  |
| 5 | ATM | Cecum-Appendix | 0.969 | 0.111 |  |  |
|  |  | Ascending-Transverse | 1.917 | 2.071 |  |  |
|  |  | Descending-Rectum | 3.467 | 0.252 |  |  |
|  |  | Total | 6.353 | 2.434 | 38.313 |  |
| 6 | ATM | Cecum-Appendix | 1.926 | 13.750 |  |  |
|  |  | Ascending-Transverse | 10.063 | 0.805 |  |  |
|  |  | Descending-Rectum | 16.766 | 0.042 |  |  |
|  |  | Total | 28.755 | 14.597 | 50.763 |  |
| 7 | ATM | Cecum-Appendix | 15.190 | 0.057 |  |  |
|  |  | Ascending-Transverse | 12.429 | 2.218 |  |  |
|  |  | Descending-Rectum | 7.071 | 0.105 |  |  |
|  |  | Total | 34.690 | 2.380 | 6.861 |  |
| 8 | Placebo | Cecum-Appendix | 5.926 | 1.213 |  |  |
|  |  | Ascending-Transverse | 19.643 | 142.012 |  |  |
|  |  | Descending-Rectum | 76.628 | 16.800 |  |  |
|  |  | Total | 102.197 | 160.025 | 156.585 |  |
| 9 | Placebo | Cecum-Appendix | 4.121 | 0.000 |  |  |
|  |  | Ascending-Transverse | 3.120 | 0.645 |  |  |
|  |  | Descending-Rectum | 0.848 | 0.098 |  |  |
|  |  | Total | 8.089 | 0.743 | 9.185 |  |
| 10 | Placebo | Cecum-Appendix | 2653.100 | 162.200 |  |  |
|  |  | Ascending-Transverse | 50.000 | 1.500 |  |  |
|  |  | Descending-Rectum | 140.500 | 0.000 |  |  |
|  |  | Total | 2843.600 | 163.700 | 5.757 |  |
| 11 | ATM | Cecum-Appendix | 4279.070 | 18.621 |  |  |
|  |  | Ascending-Transverse | 376.471 | 8.031 |  |  |
|  |  | Descending-Rectum | 3.421 | 2.000 |  |  |
|  |  | Total | 4658.962 | 28.652 | 0.615 |  |
| 12 | Placebo | Cecum-Appendix | 18.750 | 1.775 |  |  |
|  |  | Ascending-Transverse | 63.226 | 0.140 |  |  |
|  |  | Descending-Rectum | 3.920 | 15.862 |  |  |
|  |  | Total | 85.896 | 17.777 | 20.696 |  |
| 13 | Placebo | Cecum-Appendix | 329.412 | 8.333 |  |  |
|  |  | Ascending-Transverse | 18974.360 | 0.012 |  |  |
|  |  | Descending-Rectum | 11.168 | 0.012 |  |  |
|  |  | Total | 19314.940 | 8.357 | 0.043 |  |
| 14 | ATM | Cecum-Appendix | 4.571 | 0.208 |  |  |
|  |  | Ascending-Transverse | 2.154 | 0.037 |  |  |
|  |  | Descending-Rectum | 0.370 | 0.582 |  |  |
|  |  | Total | 7.095 | 0.827 | 11.656 |  |
| 15 | ATM | Cecum-Appendix | 15.143 | 0.695 |  |  |
|  |  | Ascending-Transverse | 4.839 | 0.019 |  |  |
|  |  | Descending-Rectum | 7.059 | 0.471 |  |  |
|  |  | Total | 27.041 | 1.185 | 4.382 |  |
| 16 | Placebo | Cecum-Appendix | 9.714 | 0.217 |  |  |
|  |  | Ascending-Transverse | 1.422 | 0.046 |  |  |
|  |  | Descending-Rectum | 0.215 | 4.520 |  |  |
|  |  | Total | 11.351 | 4.783 | 42.137 |  |
| 17 | ATM | Cecum-Appendix | 13.478 | 0.124 |  |  |
|  |  | Ascending-Transverse | 3.537 | 0.060 |  |  |
|  |  | Descending-Rectum | 2.069 | 2.708 |  |  |
|  |  | Total | 19.084 | 2.892 | 15.154 |  |
| 18 | Placebo | Cecum-Appendix | 3.182 | 1.073 |  |  |
|  |  | Ascending-Transverse | 3.846 | 5.225 |  |  |
|  |  | Descending-Rectum | 0.267 | 0.881 |  |  |
|  |  | Total | 7.295 | 7.179 | 98.410 |  |
| 19 | ATM | Cecum-Appendix | 0.108 | 0.376 |  |  |
|  |  | Ascending-Transverse | 2.474 | 2.189 |  |  |
|  |  | Descending-Rectum | 0.108 | 0.543 |  |  |
|  |  | Total | 2.690 | 3.108 | 115.539 |  |
| 20 | ATM | Cecum-Appendix | 3.862 | 0.542 |  |  |
|  |  | Ascending-Transverse | 42.000 | 1.867 |  |  |
|  |  | Descending-Rectum | 0.014 | 0.027 |  |  |
|  |  | Total | 45.876 | 2.436 | 5.310 |  |
| * (Total number of *F. varium*/β-actin at 3 months after treatment) / (Total number of *F. varium*/β-actin at study entry). ** Total number of *F. varium*/β-actin from cecum-appendix, ascending-transverse, descending-rectum. | | | | | |  |
